# Targeting PUF60 prevents tumor progression by retarding mRNA decay of oxidative phosphorylation in ovarian cancer

**DOI:** 10.1007/s13402-023-00859-w

**Published:** 2023-08-26

**Authors:** Cancan Zhang, Xiaoge Ni, Chunlin Tao, Ziyang Zhou, Fengmian Wang, Fei Gu, Xiaoxiao Cui, Shuheng Jiang, Qing Li, Huan Lu, Dongxue Li, Zhiyong Wu, Rong Zhang

**Affiliations:** 1 Department of Obstetrics and Gynecology, Fengxian Hospital, The Third School of Clinical Medicine, Southern Medical University, 6600 Nanfeng Road, Shanghai, 201499 China; 2 Department of Obstetrics and Gynecology, Ruijin Hospital, Shanghai Jiao Tong University School of Medicine, 197 Ruijin Er Road, Shanghai, 200025 China; 3 State Key Laboratory of Oncogenes and Related Genes, Shanghai Cancer Institute, Renji Hospital, Shanghai Jiao Tong University School of Medicine, 800 Dongchuan Road, Shanghai, 200240 China; 4 Gynecology Department, Shanghai Obstetrics and Gynecology Hospital of Fudan University, No. 419 Fangxie Road, Shanghai, 200011 China; 5 Shanghai Geriatric Medical Center, Shanghai, China

**Keywords:** Ovarian cancer (OC), PUF60, Oxidative phosphorylation, mRNA decay, PABPC1

## Abstract

**Purpose:**

Ovarian cancer (OC) is the leading cause of death from gynecological malignancies, and its etiology and pathogenesis are currently unclear. Recent studies have found that PUF60 overexpressed in various cancers. However, the exact function of PUF60 in global RNA processing and its role in OC has been unclear.

**Methods:**

The expression of PUF60 and its relationship with clinical characteristics were analyzed by multiple database analysis and immunohistochemistry. Phenotypic effects of PUF60 on ovarian cancer cell proliferation and metastasis were examined by in vitro cell proliferation assay, migration assay, and in vivo xenograft models and lung metastasis models. RNA immunoprecipitation, seahorse analyses, RNA stability assay were used to study the effect of PUF60 on the stability of oxidative phosphorylation (OXPHOS)-related genes in OC.

**Results:**

We report PUF60 is highly expressed in OC with frequent amplification of up to 33.9% and its upregulation predicts a poor prognosis. PUF60 promotes the proliferation and migration of OC cells both in vitro and in vivo. Mechanistically, we demonstrated that silencing of *PUF60* enhanced the stability of mRNA transcripts involved in OXPHOS and decreased the formation of processing bodies (P-bodies), ultimately elevating the OXPHOS level.

**Conclusion:**

Our study unveils a novel function of PUF60 in OC energy metabolism. Thus, PUF60 may serve as a novel target for the treatment of patients with OC.

**Supplementary information:**

The online version contains supplementary material available at 10.1007/s13402-023-00859-w.

## Introduction

Ovarian cancer (OC) is one of the deadliest gynecological cancers and the leading cause of gynecological cancer-related deaths worldwide. Despite continuous optimization of current treatment modalities over the past decade, the five-year overall survival (OS) rate for patients with this disease remains at a dismal 30% [1–3].Therefore, it is urgent to explore its molecular mechanism and identify the new targets for early diagnosis and treatment of OC.

Poly(U) binding splicing factor 60 (PUF60), also known as FUSE-binding Protein-interacting repressor or Ro-binding protein 1(Ro-bp1), is a nucleic acid-binding protein [4]. PUF60 directly binds to RNA and DNA and is involved in multiple nuclear processes, such as pre-mRNA splicing and transcriptional regulation [5, 6]. PUF60 is mainly composed of three domains, with two RNA-recognition motifs at the center and a U2AF homology motif (UHM) at the C-terminus. Unlike other splicing factors, the N-terminus of PUF60 lacks arginine/serine-rich (RS) and U2AF homology motif ligand motif domains [7]; therefore, PUF60 often conjuncts with U2AF to facilitate the binding of pre-mRNA binding to U2 small nuclear ribonucleoprotein. Moreover, the relative abundance of PUF60 influences the choice of alternative splice sites [8]. It has been reported that PUF60 is overexpressed in various cancers, including bladder cancer [9], colon cancer [10], hepatocellular carcinoma [11], non-small cell lung cancer [12], breast cancer [13, 14], esophageal cancer [15] and renal cell carcinoma [16], and its overexpression is closely related to its development and progression. Studies have shown through integrated copy number and expression analysis that PUF60 may be a novel potential driver [17]. However, the exact function of PUF60 in global RNA processing and its role in OC has been unclear.

Messenger RNA (mRNA) degradation and mRNA translation are two critical steps in the regulation of gene expression, with mRNA stability affecting mRNA levels, which in turn affects protein export [18–20]. Furthermore, mRNA degradation mainly includes three mechanisms: (1) deadenylation-dependent mRNA decay is the main pathway, which begins with the shortening of the poly(A) tail; (2) nonsense-mediated mRNA decay (NMD), which degrades nonsense mutated-mRNA and prevents the production of abnormal proteins to ensure normal function and activity; (3) endonucleolytic cleavage, in which site-specific RNases induce internal cleavage to produce RNA fragments, which are then degraded by exonucleases [21]. Deadenylation is a major step that triggers mRNA decay and repression of mRNA translation, resulting in a reduction in protein production [22]. The regulation of mRNA decay in tumors is complex. On the one hand, cancer cells utilize the decay mechanism to suppress the expression of tumor suppressor genes, and on the other hand, cancer cells suppress the decay mechanism to adapt to their microenvironment [23].

Processing bodies (P-bodies) are dynamic cytoplasmic RNP (ribonucleoprotein) granules that contain nontranslatable mRNAs in complex with proteins involved in translation repression and mRNA decay in eukaryotic cells [23]. P-bodies are conserved in eukaryotes and share similarities with other RNP granules, such as Cajal bodies and stress granules, and their formation relies on a complex network of protein-RNA interactions, low-complexity protein sequences and liquid-liquid phase separations (LLPS) [24]. Furthermore, P-bodies contain several proteins that participate in mRNA decay, such as decay factors UPF1, SMG6, SMG5, BRF1, BRF2 [25]; translation regulators eIF-3, eRF3, RAP55; decapping enzymes DCP2, DCP1A [26]; deadenylation factor Ccr4-NOT complex, TOB2 [27], etc. These components collaborate to regulate mRNA decay and storage.

In this study, we discovered that PUF60 expression was significantly increased in OC, and its overexpression promoted the OC cell proliferation and migration in vitro and in vivo. Further studies revealed that PUF60 promoted the decay of mRNA transcripts in OXPHOS by interacting with PABPC1, ultimately reduced the OXPHOS level. Besides, we discovered that PUF60 was a component of P-bodies, and knocking down of PUF60 decreased the P-bodies formation. Collectively, our results indicate that PUF60 might be a novel therapeutic target for patients with OC.

## Materials and methods

### Cell Culture and reagents

Human OC cell lines OVCAR8, ES-2, HO-8910PM, SKOV3, CAOV3, MCAS, COV318, FUOV-1, OVCAR3, OVCAR5, human normal ovary cell line IOSE-80, human embryonic kidney 293T (HEK293T) cells were all preserved in Shanghai Cancer Institute, Ren Ji Hospital, School of Medicine, Shanghai Jiao Tong University. Human ovarian cancer cell lines OVCAR8, HO-8910PM, SKOV3, CAOV3, MCAS, OVCAR5 and IOSE-80 were cultured in RPMI 1640 containing 10% fetal bovine serum (FBS), 2 mM glutamine and 1% penicillin/streptomycin (P/S). OVCAR3, COV318 and FUOV-1 were cultured in RPMI 1640 containing 20% fetal bovine serum (FBS), 2 mM glutamine and 1% (P/S). ES-2 and HEK293 were cultured in Dulbecco’s modified Eagle’s medium containing 10% fetal bovine serum (FBS) and 1% P/S. All cells were incubated at 37 °C in a humidified atmosphere containing 5% CO2.

### siRNA transfection

Cells were plated at 50–60% confluence in 6 well cell culture plates. OVCAR8 and ES-2 were transfected with si-PUF60 or with control siRNA. The sequences of the siRNA used were as follows: si-PUF60-1, sense (5’-3’): GCUACGGCUUCAUUGAGUATT, antisense (5’-3’): UACUCAAUGAAGCCGUAGCTT; si-PUF60-2, sense (5’-3’): CAGAAAUCAUUGUCAAGAUTT, antisense (5’-3’): AUCUUGACAAUGAUUUCUGTT. SiRNA oligos were purchased by Gene Pharma (Shanghai, China). Transfection steps were performed according to the reagent operation manual of Lipofectamine® RNAiMAX (Thermo Fisher Scientific, Waltham, MA, USA).

### RNA isolation and quantitative real-time PCR

Total cellular RNA was extracted using Trizol reagent (Takara). PrimeScript RT-PCR kit (Takara) was used to perform the RT according to the protocol. Real-time PCR was used to determine the mRNA expression on a 7500 real-time PCR system (Applied Biosystems) according to the manual.

of SYBR Green qPCR Master Mix (Bimake). Data were normalized to 18s RNA expression and represented as the average of three repeated experiments. Prime sequences used for PUF60, ATP5J2, ATP5L, ATP6V0C, ATP6V0E1, NDUFS8, NDUFA1, NDUFA2, NDUFA8, NDUFC2, NDUFS5, NDUFS6, COX7C, UQCRQ and 18s detection were shown in Supporting Table 1.

### RIP-Seq

OVCAR8 cells seeded in a 10 cm dish at 70–80% confluency were harvested by cell scraping. 2 µg of PUF60 antibody (ab225705, Abcam) was conjugated to protein A/G magnetic beads (Miltenyi Biotec) by incubation for at 4 °C overnight, followed by washing three times and incubation with pre-prepared cell lysate in RIP buffer (150 mM KCl, 25 mM Tris (pH 7.4), 5 mM EDTA, 0.5 mM DTT, 0.5% NP40, 1×protease inhibitor) at 4 °C overnight. After washing with RIP buffer for three times, beads were resuspended in 80 µl PBS, followed by DNA digestion at 37 °C for 15 min and incubation with 50 µg of proteinase K (Thermo Fisher) at 37 °C for 15 min. Input and co-immunoprecipitated RNAs were recovered by TRIzol for RNA-seq library construction using KAPA Library Quantification Kit (KK8401). The final library was sequenced with illumina HiSeq X 10. The mRNA levels detected in the IP fractions were normalized to their respective TL fractions(input)to compensate for changes in mRNA expression. Log2-ratios of IP vs. input values were calculated for each transcript and the value was higher than 1 indicated statistically significant difference.

### RNA-seq

Total RNAs isolated from OVCAR8 transfected with siPUF60 or control siRNA by Trizol reagent following the manufacturer’s instructions. RNA quality was assessed using an Agilent Bioanalyzer 2100 (Agilent technologies, Santa Clara, CA, US) and sent for library preparation. Total RNA was amplified, labeled, and purified by RNAClean XP Kit (Beckman Coulter, Inc., Kraemer Boulevard Brea, CA, USA) and RNase-Free DNase Set (QIAGEN, GmBH, Germany) following the manufacturer’s guidelines. Then, the purified RNA was sequenced by Illumina Hiseq X10 platform by Majorbio Genomics (Shanghai, China), followed by analyzing the sequence data using GRCm38.p10 genome database.

### Plasmid transfection

The sequences of the short hairpin (sh)RNAs targeting PUF60 were sh-1, 5′- GCTACGGCTTCATTGAGTACG-3′ and sh-2, 5′- CTGAGACTCATAAGGCCATCC-3′. The shRNA plasmids and control plasmid were purchased from GenePharma (Shanghai, China). All these plasmids were packaged into virus particles using HEK 293T cells and the viral titers were determined. Then the target cells were infected with 1 × 10^8^ lentivirus-transducing units with 6 µg/mL polybrene (Sigma-Aldrich, St. Louis, MO, USA). The infected cells were then screened with 2 µg/mL puromycin after 72 h. The efficiency of the knockdown or overexpression was verified by western blotting.

### Western blotting

 Total cellular protein and nuclear-cytosol protein were extracted using a total protein extraction buffer (Beyotime, China). Cell lysates were separated by SDS-PAGE followed by blocking in 1% BSA (Bovine Serum Albumin), then incubated with primary antibodies and species-specific secondary anti-bodies. Bound secondary antibodies were detected with the Odyssey imaging system (LI-COR Biosciences, Lincoln, NE). Primary antibodies used for PUF60, β-actin, PABPC1 detection were shown in Supporting Table 2.

### Seahorse analyses

The assays for extracellular acidification rate (ECAR) and oxygen consumption rate (OCR) in the cultured cells were performed with the Seahorse XF96 Flux Analyzer (Seahorse Bioscience, Agilent) according to the manufacturer’s instructions. Briefly, OVCAR8 and ES-2 cells were seeded in a XF96-well plate at a density of 1 × 10^4^ per well with indicated treatments. The media was replaced with assay media at 1 h before the assay. For the glycolytic stress test (Seahorse Cat. #103020-100), 10mM glucose, 1µM oligomycin and 50mM 2-deoxyglucose (2-DG) were injected to the wells. For the mitochondrial stress test (Seahorse Cat. #103015-100), 1µM oligomycin, 1µM FCCP, 0.5µM rotenone and 0.5µM antimycin A were added to the wells. Above experiments were performed in triplicate manner and repeated twice.

### RNA Stability Assay and sequencing for mRNA lifetime

Cells were treated with 10 µg/ml actinomycin D and collected at indicated time points. The total RNA was extracted by Simply P Total RNA Extraction kit (BSC52S1, BIOER) and analyzed by RT–PCR. The turnover rate and half-life of mRNA were estimated according to a previously published paper [28].

### Co-immunoprecipitation (Co-IP) assay

Protein A/G beads (Santa Cruz Biotechnology) was pre-incubated with FLAG antibody (Sigma) and IgG for 30 min on a spinning wheel. The bead-antibody complexes were then suspended with the total protein extraction. All Co-IP was performed overnight on a spinning wheel at 4 °C. The beads were washed 3 times with extraction buffer and were collected by centrifugation at 5000 rpm. The immunoprecipitants were subjected to western blotting.

### Immunofluorescence (IF)

Ovarian cancer cells were planted in 8-well chambers (Ibidi, Germany) for IF. We fixed cells with 4% polyformaldehyde (30 min), permeabilized with 0.1% TritonX-100 (10 min) and blocked with 10% BSA (1 h) at room temperature. All cells were incubated with the primary antibodies at room temperature for 2 h and then labeled with Alexa Fluor-488-conjugated Alexa (1:400, Rabbit, Sigma, USA) and Fluor-594-conjugated secondary antibody (1:400, Mouse, Sigma, USA) at room temperature. DAPI was used to stain the nucleus for 5 min (Sigma, USA). Confocal microscopy (LSM 510, METALaser scanning microscope, Zeiss) was used to acquire the images. Primary antibodies used for PUF60, PABPC1 and DCP1A detection were shown in Supporting Table 2.

### Immunohistochemistry (IHC) staining

Immunohistochemical staining were performed as described [29]. After treatment with diaminobenzidine and counterstaining with hematoxylin, all the sections were observed and photographed with a microscope (Axio Imager: Carl Zeiss). Scoring was conducted according to the ratio and intensity of positive-staining cells. The staining extent was scored as: 0–5% scored 0; 6–35% scored 1; 36–70% scored 2; more than 70% scored 3. The staining intensity was scored as: 0 (negative), 1 (weak), 2 (moderate) and 3 (strong). The immunoreactivity score (IRS) = extent score × intensity score, resulting in low (0–2) and high (3–9) values for each specimen. The final immunoreactive score was judged by two senior pathologists in a blinded manner. Primary antibodies used for PUF60, PABPC1 and DCP1A detection were shown in Supporting Table 2.

### Clinical samples

Human ovarian cancer, ovary ovarian cysts and normal ovarian surface epithelium were obtained from the Department of Obstetrics and Gynecology, Fengxian Hospital, Southern Medical University and the Department of Gynecology, Changzhou Maternal and Child Care Hospital. None of them had received radiotherapy, chemotherapy and other related anti-tumor therapies before surgery. All human tissues were obtained with informed consent and all protocols were approved by the ethical review committee of the World Health Organization Collaborating Center for Research in Human Production.

### Cell viability assay

The cells were plated in 96-well plates at a density of 3000 cells per well with 100 µl complete culture medium and cultured for 2-5days. Each group contains five wells. 10 µl Cell Counting Kit-8 (Dojindo, Japan) solution was added to each well after 24 h, 48 h, 72 and 96 h. CCK8 was metabolized to produce a colorimetric dye that was read at 450 nm using a microplate reader.

### Cell migration

2 × 10^4^ cells were seeded into the upper chamber of the transwell plate (Millipore, USA). Cells were allowed to migrate for 24 h at 37 ° C. The migrated cells were then fixed and stained with 0.1% crystal violet, six randomly selected fields were photographed, and the cell numbers were counted.

### Cell apoptosis assay

Cell apoptosis assay was performed using an Annexin V/PI apoptosis kit (BD Biosciences, Franklin Lakes, NJ, USA) following the manufacturer’s protocol. Adherent cells were cultured in serum-free medium for 24 h. The cells were detached with 0.25% trypsin (without ethylenediaminetetraacetic acid), washed, re-suspended with binding buffer, and stained. The percentages of Annexin V-positive and propidium iodide-negative cells were determined by flow cytometry using BD FACS Calibur (BD Biosciences).

### In vivo tumor xenograft model

Six-week-old female athymic nude (nu/nu) mice (SLAC, Shanghai, China) were randomly divided into four groups and injected subcutaneously in the right flank with the stable single cell clones of OVCAR8-sh and control cells, lenti-PUF60 and lenti-vector cells at 5 × 10^6^ cells in 100 µl PBS medium for each nude mouse. We measured tumor volume once a week. After mice were killed. The tumors were dissected and fixed with phosphate-buffered neutral formalin for standard histologic examination. Then paraffin embedded tumor samples were cut into 4-µm-thick sections for apoptosis detection. Then following immunohistochemistry staining PUF60, KI67, Caspase3, DCP1A, NDUFA2. Primary antibodies used for detection were shown in Supporting Table 2. The Mice were manipulated and housed according to protocols approved by the East China Normal University Animal Care Commission.

### In vivo lung metastatic model and living image

Construction of PUF60 overexpression stable cell line with the virus with a luciferase label was produced by Gene Pharma. For the in vivo metastasis model, the mice were randomly divided into two groups (vector and PUF60 groups) and injected with 2 × 10^6^ cells via the tail vein. After 60 days, a single dose of 150 mg/kg was intraperitoneally injected with D-luciferin, luciferin imaged using non-invasive bioluminescence imaging living imaging system (PerkinElmer, Waltham, MA) 10 min after the injection and analyzed using Living Image 3.0 software.

### Data mining using Oncomine, TCGA, GTEx, Kaplan Meier plotter and R2

PUF60 gene expression was analyzed using microarray gene expression datasets deposited in Oncomine database (https://www.oncomine.org). A combined filter was applied to display the corresponding datasets. The Cancer Type was defined as Ovarian Cancer and Data Type was mRNA, and Analysis Type was Cancer versus Normal Analysis. The expression levels of PUF60 gene were read from the displayed bar chart and these data were parsed into Excel to analyze. The gene expression data for ovarian adenocarcinoma was downloaded from TCGA, which were processed by Broad Institute’s TCGA work group. The gene expression data for normal ovarian was downloaded from GTEx (https://gtexportal.org). Survival rate analyzed by a Kaplan–Meier analysis of 1104 Ovarian cancer patients were referenced from an online database-The Kaplan Meier plotter (https://kmplot.com/analysis/) [30]. Another survival rate analyzed by a Kaplan–Meier analysis of 51 ovarian cancer patients were referenced from was from an online database-R2: Genomics Analysis and Visualization Platform (http://r2.amc.nl).

### Statistical analysis

Data are shown as means ± S.D. Statistical analyses were done using SPSS 20.0 for Windows (IBM). Cumulative survival time was calculated by the Kaplan-Meier method and analyzed by the log-rank test. Correlation of PUF60 expression with categorical clinical variables in patients with OC was evaluated by χ^2^ test or Fisher’s exact test. The student’s t-test or one-way ANOVA was used for comparison between groups. Values of *P* < 0.05 were considered statistically significant. A p value of less than 0.05 was considered statistically significant. Data are presented as the mean ± SD (*P < 0.05, **P < 0.01, ***P < 0.001).

## Results

### PUF60 is highly expressed in OC and predicts unfavorable outcomes in patients with OC

**Fig. 1:**
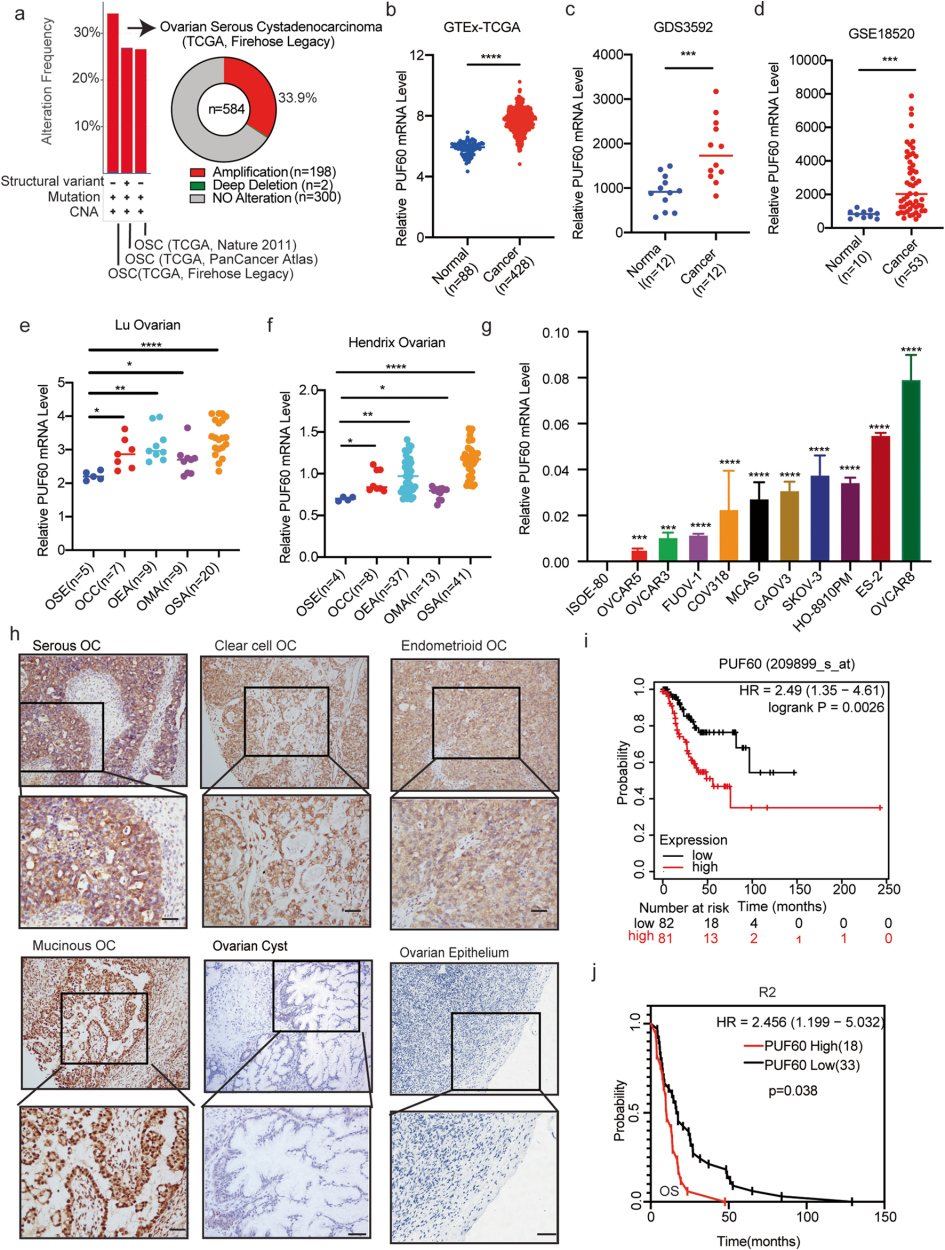
PUF60 is upregulated in OC and predicts poor prognosis. **a** Copy number alteration of PUF60 in the cBioPortal for Cancer Genomics. **b**-**f** PUF60 expression in tumors and normal tissues using TCGA, GTEx and Oncomine. OSE: Ovarian Surface Epithelium;OCC: Ovarian Clear Cell Adenocarcinoma; OEA: Ovarian Endometrioid Adenocarcinoma; OMA: Ovarian Mucinous Adenocarcinoma; OSA: Ovarian Serous Adenocarcinoma. **g** mRNA expression of PUF60 in ten different OC cell lines and one human normal ovarian epithelial cell line-ISOE-80. The p-value is a comparison between ovarian cancer cells and normal ovarian epithelial cell line-ISOE-80. **h** Representative IHC images of PUF60 in normal ovaries or ovarian cancer. Scale bar is 50 μm. **i** Kaplan-Meier survival curve (stage:I + II) of OC data from KM-plotter. **j** Kaplan-Meier survival curve of OC data from R2. Data are presented as the means ± SEM. *P < 0.05; **P < 0.01; ***P < 0.001, ****P < 0.0001

PUF60 was screened from the most amplification region in OC, so we first analyzed the copy number alteration in the cBioPortal for Cancer Genomics. As a result, PUF60 was frequently amplified up to 33.9% in OC samples (Fig. 1a). To determine PUF60 expression in OC, we first analyzed the mRNA expression level of PUF60 in GTEx and TCGA. The results indicated that the expression of PUF60 in serous ovarian tumors was significantly higher than that in the normal ovarian surface epithelium (Fig. 1b). Then, we analyzed the independent OC microarray data in the GEO database and Oncomine databases, and discovered that the mRNA expression of PUF60 in various subtypes OC tissue was significantly higher than that in normal ovarian tissue (Fig. 1c-f, Fig. S1a-c). To further verify PUF60 expression of in OC, we detected the expression of PUF60 in 10 different OC cell lines and one human normal ovarian epithelial cell line-ISOE-80 by qPCR. PUF60 mRNA expression in OC cells was showed significantly higher than that in normal ovarian epithelial cell line (Fig. 1g). Additionally, we analyzed the PUF60 expression in other malignant tumors and discovered that PUF60 is highly expressed in lymphoid neoplasm diffuse large B-cell lymphoma (DLBC), glioblastoma multiforme (GBM), brain lower grade glioma (LGG), liver hepatocellular carcinoma (LIHC), pancreatic adenocarcinoma (PAAD), thymoma (THYM) and uterine carcinosarcoma (UCS) (Fig. S1d).

To further evaluate the PUF60 protein expression in OC, immunohistochemistry (IHC) was performed on 281 OC tissues and 112 ovarian cyst tissues, and the results showed that the PUF60 expression in OC was significantly higher than that in benign ovarian serous cystadenoma (Fig. 1h). This finding was consistent with our analysis of PUF60 mRNA expression in public databases. Furthermore, we examined the correlation between the PUF60 expression status and clinicopathological characteristics of 281 patients with OC. The results indicated that the expression level of PUF60 was closely associated with age, clinical stages and subtypes (Table 1), with higher expression in older patients and highly malignant subtypes, such as in advanced OC, clear-cell OC, and high-grade serous OC.

**Table 1:** Correlation of the clinicopathological parameters with PUF60 expression

Catalog	Level	PUF60 low	PUF60 high	Total	X^2^	P
Classification						
	Ovarian Cyst	65(58.04%)	47(41.96%)	112	5.284	0.022
	Epithelial Ovarian Cancer	127(45.20%)	154(54.80%)	281		
Age						
	< 50y	54(60.00%)	36(40.00%)	90	6.403	0.011
	≥ 50y	93(48.69%)	118(51.31%)	191		
Stage						
	I + II	80(51.28%)	76(48.72%)	156	5.245	0.022
	III + IV	47(37.60%)	78(62.40%)	125		
Type						
	High-grade Serous OC	42(33.87%)	82(66.13%)	124	36. 823	< 0.001
	Low-grade Serous OC	31(70.45%)	13(29.55%)	44		
	Clear cell OC	13(26.00%)	37(74.00%)	50		
	Endometrioid OC	15(53.57%)	13(46.43%)	28		
	Mucinous OC	26(74.29%)	9(25.71%)	35		
Lymph metastasis						
	Positive	30(51.72%)	28(48.28%)	58	1.258	0.262
	Negative	97(43.50%)	126(56.50%)	223		
	Total	127	154	281		

Based on the association of high PUF60 high expression with aggressive tumor behaviors, PUF60 may serve as a novel potential prognostic marker. Thus, we assessed the correlation between PUF60 expression and clinical follow-up information using the Kaplan–Meier plotter. As a result, we discovered that patients with high PUF60 expression had significantly shorter progression-free survival (PFS) in early-stage OC patients, but no difference in advanced-stage patients (Fig. 1i, Fig. S1e-f). We also analyzed the effect of PUF60 expression level on patient’s prognosis in R2 dataset. Patient with high PUF60 expression tend to have poor overall survival (OS) (Fig. 1j). Collectively, these results indicate that PUF60 is upregulated in OC, acts as an indicator of OC progression, and predicts poor prognosis.

### PUF60 promotes proliferation, invasion, and apoptosis inhibition of OC cells in vitro

**Fig. 2:**
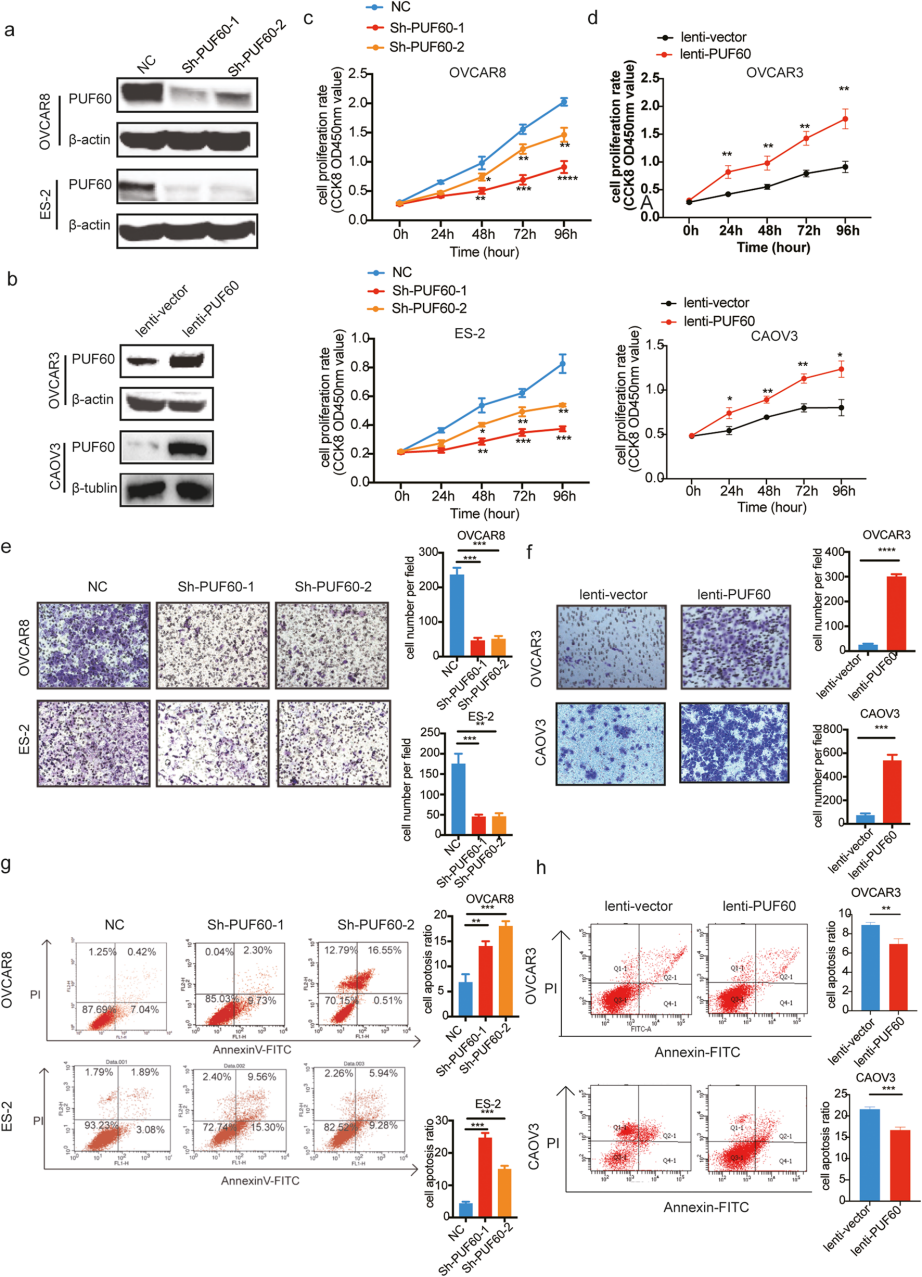
PUF60 promotes proliferation and invasion and apoptosis inhibition of OC cells in vitro. **a** Interference efficiency verification of PUF60 in OVCAR8 and ES-2 cells. Left: western blot gels, right: Protein quantification, interference group values were compared with the control group. **b** Overexpression efficiency verification of PUF60 in OVCAR3 and CAOV3 cells. Left: western blot gels, right: Protein quantification, interference group values were compared with the control group. **c** Relative cell viability of OVCAR8 and ES-2 cells stably expressing shNC or shPUF60. **d** Relative cell viability OVCAR3 and CAOV3 stably expressing lenti-vector or lenti-PUF60. **e** Cellular migration ability was detected by transwell migration in OVCAR8 and ES-2 cells expressing shNC, shPUF60, representative pictures on the left and the number of migrated cells on the right. **f** Cellular migration ability was detected by transwell migration in OVCAR3 and CAOV3 stably expressing lenti-vector, lenti-PUF60, representative pictures on the left and the number of migrated cells on the right. **g** Flow cytometry for detection of apoptosis by Annexin/PI double staining in OVCAR8 and ES-2 cells expressing shNC and shPUF60. **h** Flow cytometry for detection of apoptosis by Annexin/PI double staining in OVCAR3 and CAOV3 cells expressing lenti-vector and lenti-PUF60. Data are presented as the means ± SEM. *P < 0.05; **P < 0.01; ***P < 0.001, ****P < 0.0001

To elucidate the biological functions of PUF60 in OC, we conducted a series of gain-of-function and loss-of-function studies in OC cells. OVCAR8 and ES-2 showed relatively high PUF60 expression, while OVCAR3 and CAOV3 showed relatively low PUF60 expression (Fig. 1g), so we selected OVCAR8 and ES-2 cells for stable knockdown while OVCAR3 and CAOV3 cells were selected for artificial upregulation of PUF60 expression respectively (Fig. 2a-b*)*. We determined the effect of PUF60 on the proliferation of OC cells through CCK-8 assays. Results indicated that compared with the control group, OVCAR8 and ES-2 were significantly inhibited after PUF60 knockdown, while OVCAR3 and CAOV3 proliferation were significantly promoted after PUF60 overexpression (Fig. 2c-d*)*. We also examined the effect of PUF60 on the migration of OC cells via transwell migration assays. The findings revealed that compared with the control group, PUF60 knockdown significantly reduced migratory abilities, while PUF60 overexpression significantly increased migratory abilities (Fig. 2e-f). Additionally, we explored the effects of PUF60 on OC cell apoptosis by flow cytometry. The apoptosis rates of PUF60 knockdown groups in OVCAR8 and ES-2 were significantly higher, while PUF60 overexpressed groups in OVCAR3 and COAV3 were significantly lower than that in the control group, indicating that PUF60 also inhibited cell apoptosis (Fig. 2g-h).

### PUF60 promotes tumor growth and metastasis of OC cells in vivo

**Fig. 3:**
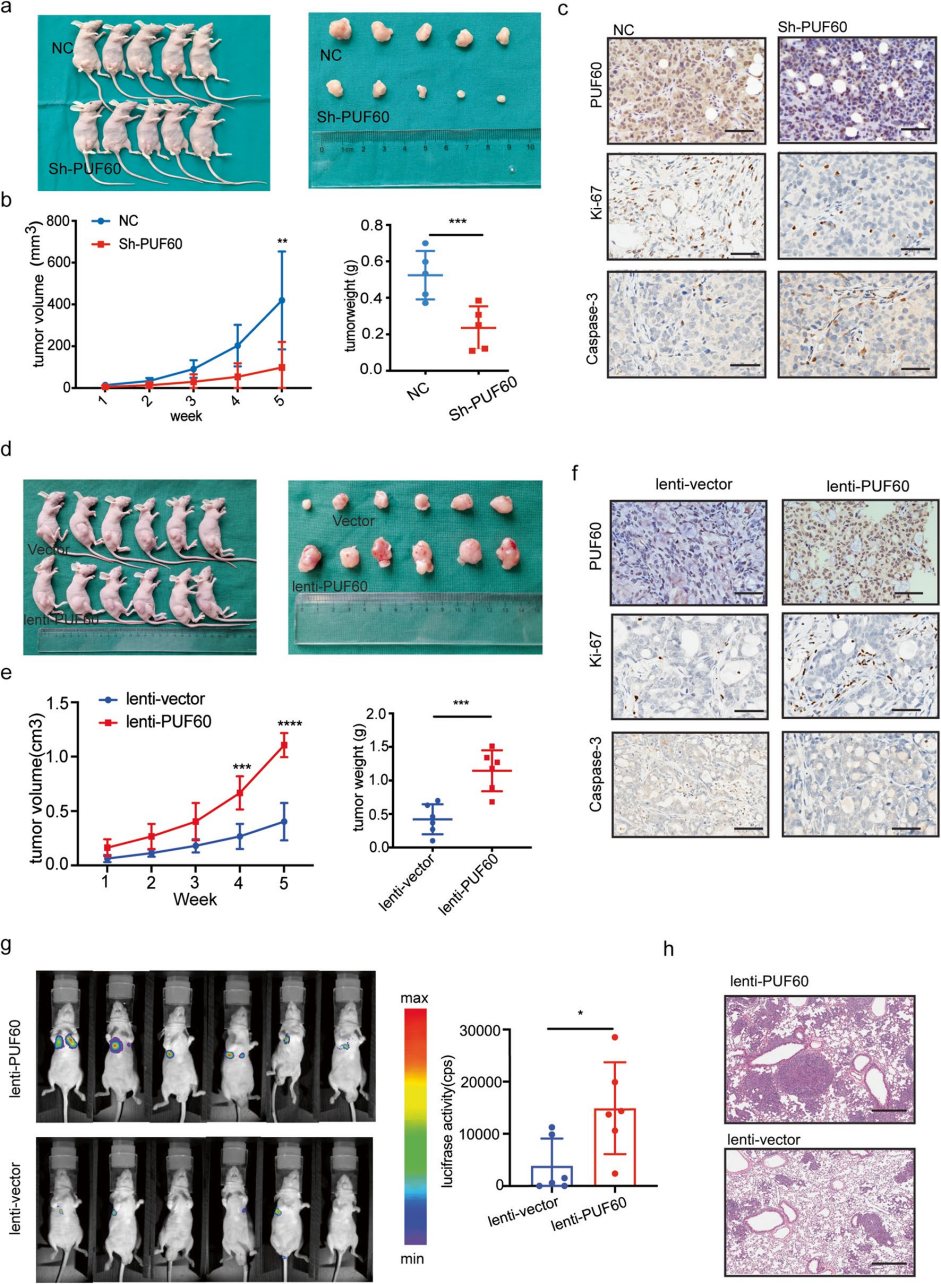
PUF60 promotes subcutaneous xenografts growth and lung metastases in mice. **a**-**c** Subcutaneous xenografts transplanted with OVCAR8 cells expressing shNC and shPUF60 (*n* = 5). Representative IHC images of PUF60, ki-67, Caspase3 from subcutaneously transplanted samples, scale bar is 50 μm. **d**-**f** Subcutaneous xenografts transplanted with OVCAR3 cells expressing lenti-vector, lenti-PUF60 (*n* = 6). Representative IHC images of PUF60, Ki-67, Caspase3 from subcutaneously transplanted samples, scale bar is 50 μm. **g**-**h** Representative living image of mice injected with luciferase expressing OVCAR8 cells through the tail vein. Representative HE images show the size of metastases in the lungs, scale bar is 1 mm

A subcutaneous xenograft model was established to explore the effects of PUF60 expression on tumors in vivo. The stable PUF60 knockdown and control OVCAR8 cells were subcutaneously injected into the nude mice. The stable PUF60-knockdown group showed a delay in the growth speed of tumors, as well as reduced tumor weight and volume (Fig. 3a-b). Furthermore, IHC staining revealed decreased Ki67expression and increased Caspase 3 expression in the xenografted tumors from PUF60-knockdown cells compared with those from control cells (Fig. 3c). Similar experiments were performed to explore the effects of PUF60 overexpression on tumor growth in subcutaneous xenografts. An obvious stimulation in the growth speed of tumors as well as increased tumor weight and volume were observed in the group stably overexpressing PUF60 (Fig. 3d-e). Additionally, IHC staining revealed increased Ki67 expression and decreased Caspase 3 expression in the xenografted tumors from PUF60 overexpressing cells compared with those from control cells (Fig. 3f).

A lung metastasis model was established to explore the impacts of PUF60 expression on tumors in vivo. OVCAR8 cells transfected with Vector-Luc and PUF60-Luc were injected into the tail veins of BALB/C nude mice. Thirty days after injection, the tumor metastasis status was examined using an imaging system that detects luciferase signals. The representative bioluminescent images of the different groups are shown in Fig. 3g, the luciferase signal of metastatic lung nodules in the PUF60-overexpressing group was larger than that in the control group. Furthermore, hematoxylin and eosin staining of lung tissue confirmed that the incidence of metastasis in the lung significantly increased in the PUF60-overexpressing group than in the control group (Fig. 3h).

### PUF60 reduces the OXPHOS level and elevates glycolysis level in OC cells

**Fig. 4:**
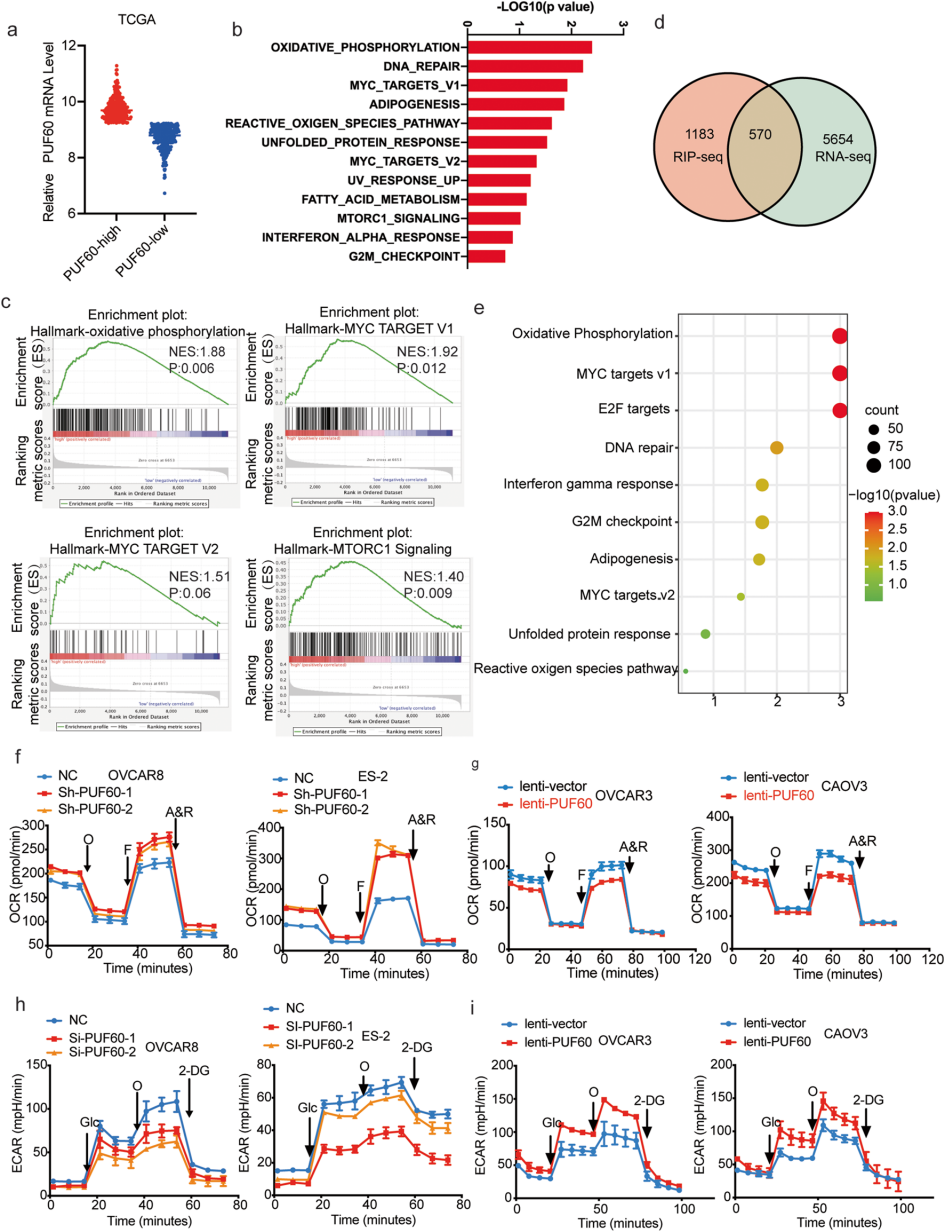
PUF60 reduces the OXPHOS level and elevates glycolysis level in OC cells. **a** PUF60 expression levels in PUF60-high samples compared with their low-PUF60 counterparts among 585 OC patients in TCGA (PUF60 high group, *n* = 293; PUF60 low group, *n* = 292). **b** GSEA of specimens with high and low expression of PUF60 based on the data from TCGA. Top 12 gene sets are upregulated in PUF60 high phenotype. **c** Representative gene sets are upregulated in PUF60 high phenotype. (NES, normalized enrichment score.). **d** Overlap of PUF60 target genes identified by RIP–seq and RNA-Seq data. **e** Top 10 KEGG pathways of 570 target genes. **f**-**i** Mitochondrial stress test and glycolytic function of OVCAR8 and ES-2 cell lines versus PUF60 knockdown and OVCAR3 and CAOV3 cell lines versus PUF60 overexpression as measured by the extracellular acidification rate (ECAR, *n* = 3) and the oxygen consumption rate (OCR, *n* = 3). Glc: Glucose; O: Oligomycin; F: FCCP, A&R: Antimycin A and Rotenone

To explore the mechanism by which PUF60 mediated cell survival, we divided OC samples of the TCGA database into two groups based on median PUF60 expression (Fig. 4a). Gene set enrichment analysis (GSEA) of two group transcriptome data using the Hallmarks gene sets revealed striking alterations in metabolic processes, including OXPHOS, Myc targets database, and mTORC1 signaling (Fig. 4b-c). To gain insight into the underlying mechanism of PUF60 in promoting OC development, we sequenced purified RNA from PUF60-RNA-binding protein immunoprecipitation (RIP) samples and discovered that PUF60 could bind directly to1753 mRNAs. Simultaneously, we performed RNA sequencing of PUF60-knockdown and control cells and found 6224 differentially expressed genes. Combined with our PUF60-RIP-seq and RNA-seq data, 570 mRNAs were identified as PUF60 binding targets (Fig. 4d). The Kyoto Encyclopedia of Genes and Genomes (KEGG) pathway analyses of 570 target genes were performed using DAVID database. Results revealed that the most significantly enriched KEGG pathway was OXPHOS (Fig. 4e), which was consistent with the results of the previous GSEA. The above findings indicate that PUF60 might play a crucial role in OXPHOS.

To verify the effect of PUF60 on tumor metabolism, we examined the oxygen consumption rate (OCR; an indicator of OXPHOS) and extracellular acidification rate (ECAR; an indicator of glycolysis) using the Seahorse XF96 analyzer. The results showed that PUF60 knockdown increased OXPHOS levels and decreased glycolysis levels in OVCAR8 and ES-2 cells, while overexpression of PUF60 led to opposite results (Fig. 4f-i, Fig. S3a-d). Collectively, these findings indicate that PUF60 maintains the metabolic balance of OC cells by regulating the level of OXPHOS and glycolysis.

### PUF60 accelerates the mRNA decay of OXPHOS-related genes in OC

**Fig. 5:**
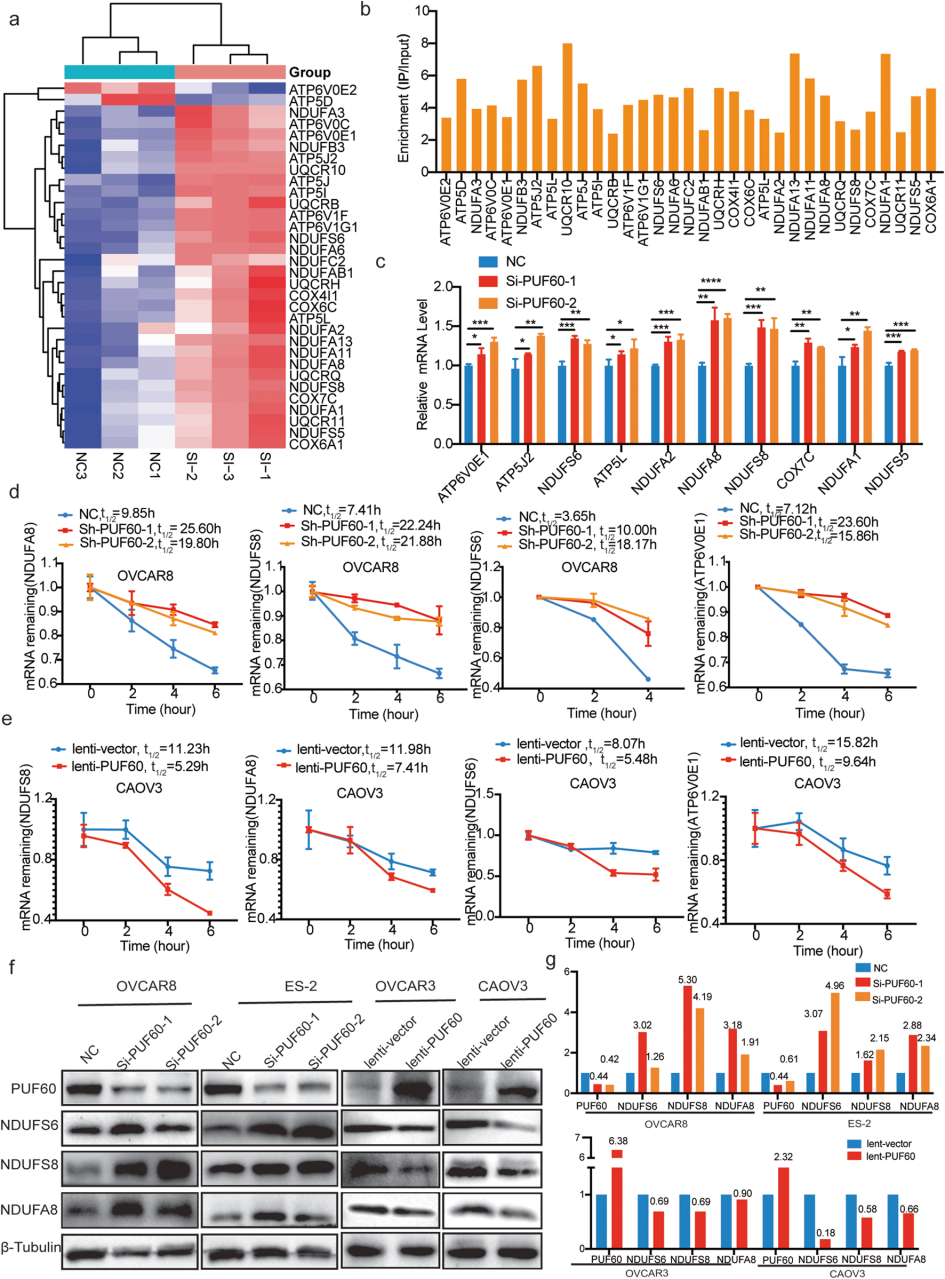
PUF60 promotes mRNA decay of genes related to OXPHOS. **a** Heat map of OXPHOS related genes using our RNA-Seq data. **b** Enrichment of PUF60 binding RNA of representative OXPHOS related genes using RIP–seq data. **c** Relative mRNA expression of representative OXPHOS related genes in control and PUF60 knockdown OVCAR8 cells. **d** mRNA half-life measurement of representative OXPHOS related genes in control and PUF60 knockdown OVCAR8 cells. **e** mRNA half-life measurement of representative OXPHOS related genes in control and PUF60 overexpressed CAOV3 cells. **f** Relative protein expression of OXPHOS related genes in control and PUF60 knockdown OVCAR8 and ES-2 cells, and in control and PUF60 overexpressed OVCAR3 and CAOV3 cells. **g** Quantitative analysis of protein bands in Fig. 5f. Data are presented as the means ± SEM. *P < 0.05; **P < 0.01; ****P < 0.0001

To further understand how PUF60 inhibits OXPHOS, we examined the expression levels of 32 target genes enriched in the OXPHOS in OC cells and found that 30 genes of them were upregulated in the PUF60-knockdown group (Fig. 5a). Our RIP-seq data indicated that the PUF60 protein bind to these 32 target transcripts (Fig. 5b). Quantitative real-time PCR data further verified that PUF60 reduced the mRNA expression of target genes enriched in the OXPHOS (Fig. 5c).

RNA-binding proteins (RBPs) are a critical group of multifunctional proteins that precisely regulate all aspects of gene expression, from alternative splicing to mRNA trafficking, stability, and translation [31]. Thus, we performed mRNA stability measurements in PUF60-knockdown and control cells. The half-life of target mRNAs was significantly increased to approximately 2–5 times in PUF60-knockdown cells than that in control cells (Fig. 5d), while reduced by 50% approximately in PUF60-overexpressed cells than that in control cells (Fig. 5e). Therefore, PUF60 reduced the expression of OXPHOS-related mRNAs by promoting the mRNA decay, ultimately reducing the OXPHOS level of OC cells. Because mRNA decay directly affects protein output, so we measured the protein expression of target genes enriched in OXPHOS. NDUFA8, NDUFS8, and NDUFS6 were decreased in PUF60-knockdown tissue and increased in PUF60-overexpression tissues (Fig. 5f-g). IHC staining displayed consistent results (Fig. S2a-d), which was consistent with previous observations of mRNA levels in OC cells.

### PUF60 promotes target mRNAs decay by interacting with PABPC1 in OC

**Fig. 6:**
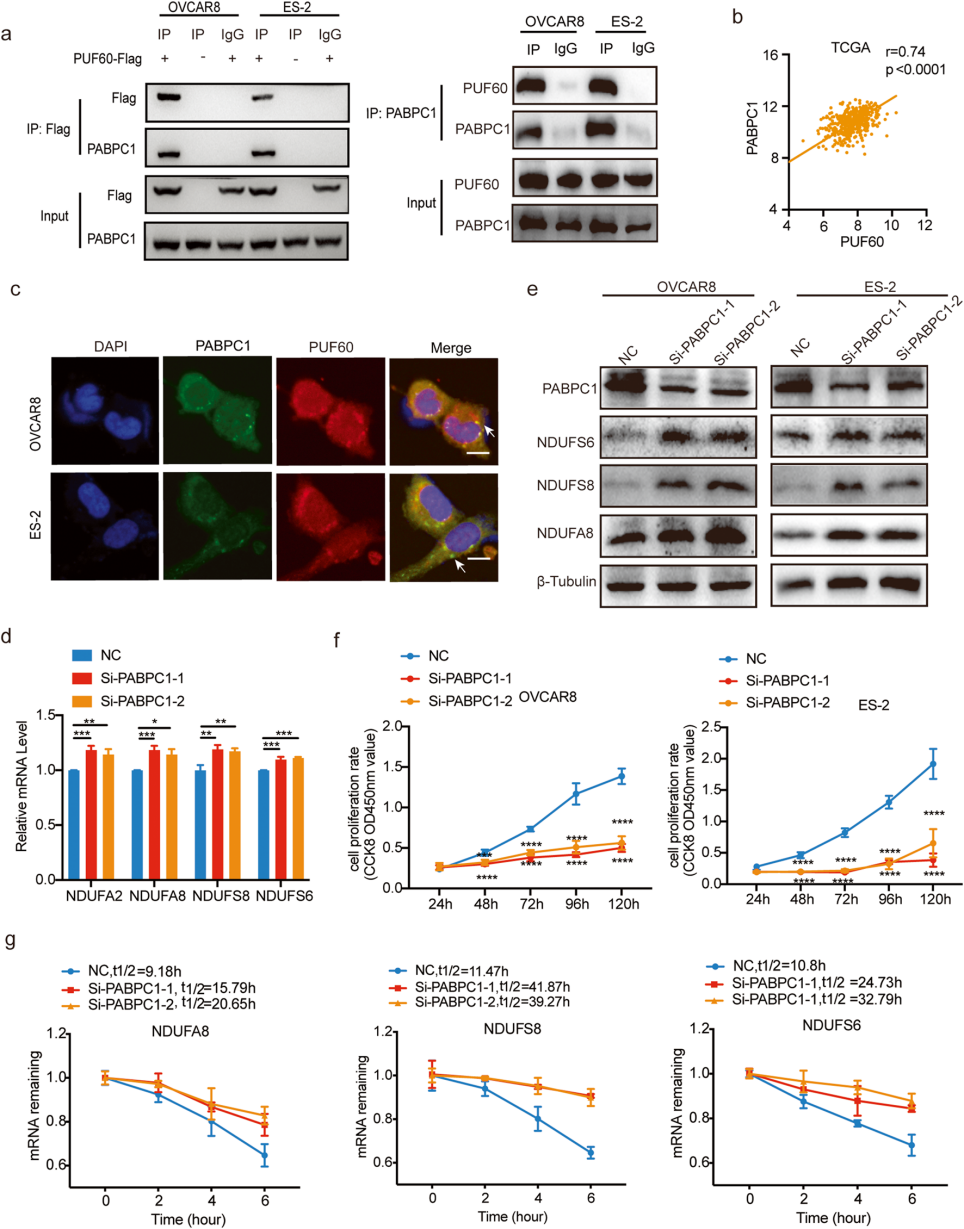
PUF60 promotes target mRNAs decay by interacting with PABPC1 in OC. **a** Co-immunoprecipitation of PUF60 and PABPC1in OVCAR8 and ES-2 cells. PUF60-Flag: Cells transfected with PUF60-Flag plasmid. **b** The correlation analysis of PUF60 and PABPC1 in OC samples from TCGA. **c** Immunofluorescence of PUF60 and PABPC1 in OVCAR8 and ES-2 cells. Scale bar is 10 μm. **d**-**e** Relative mRNA expression and protein of representative OXPHOS related genes in control and PABPC1 knockdown OVCAR8 cells. **f** Relative cell viability of control and PABPC1 knockdown OVCAR8 and ES-2 cells. **g** mRNA half-life measurement of representative OXPHOS related genes in control and PABPC1 knockdown OVCAR8 cells. Data are presented as the means ± SEM. *P < 0.05; **P < 0.01; ****P < 0.0001

To unveil the underlying mechanism by which PUF60 modulates mRNA decay, we searched for interaction partners of PUF60 from the BIOGRID database and discovered that poly(A)-binding protein cytoplasmic 1 (PABPC1), a known gene that promotes mRNA decay [32, 33], might interact with PUF60. Then, we performed a co-immunoprecipitation assay and discovered that overexpressed Flag-PUF60 physically interacted with endogenous PABPC1 (Fig. 6a). We found a positive correlation between the mRNA expression of PUF60 and PABPC1 in OC samples from the TCGA (Fig. 6b). Immunofluorescence assays revealed that PUF60 colocalized with PABPC1 in both OVCAR8 and ES-2 cells (Fig. 6c). In addition, we found that PABPC1 interference increased the expression of OXPHOS genes (Fig. 6d-e), inhibited the proliferation of OC cells (Fig. 6f), and inhibited the degradation of related genes (Fig. 6g), these results were consistent with PUF60 interference. Collectively, PUF60 promoted target mRNAs decay by interacting with PABPC1.

### PUF60 promotes the formation of P-bodies in OC

**Fig. 7:**
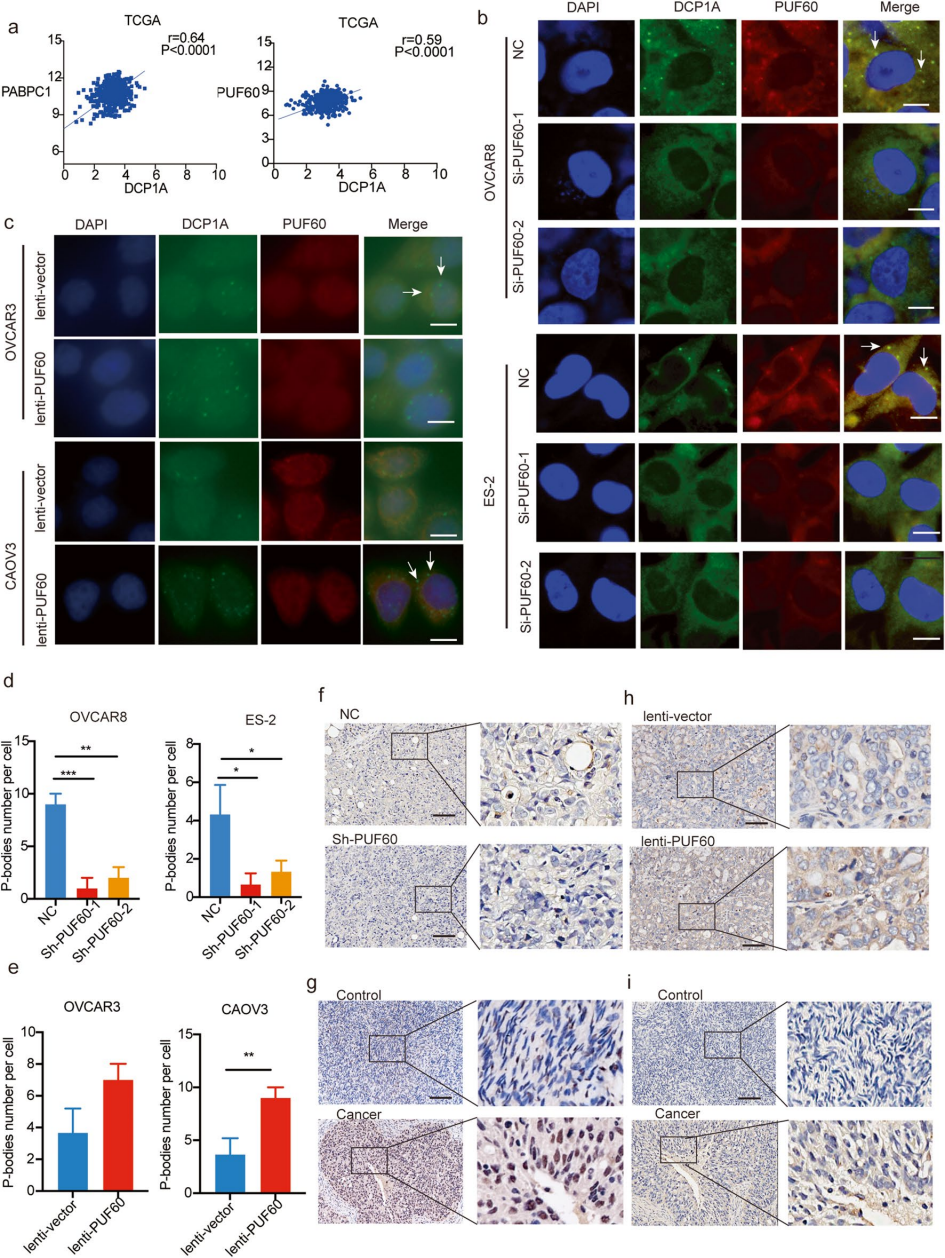
PUF60 promotes the formation of P-bodies in OC. **a** The correlation analysis of PUF60/PABPC1 and DCP1A in OC samples from TCGA. The correlation analysis of PUF60 and DCP1A in OC samples from TCGA. **b**-**c** Immunofluorescence of PUF60 and DCP1A in control and PUF60-knockdown OVCAR8 and ES-2 cells. Scale bar is 10 μm. **b** Immunofluorescence of PUF60 and DCP1A in control and PUF60-knockdown OVCAR8 and ES-2 cells. Scale bar is 10 μm. **c** Immunofluorescence of PUF60 and DCP1A in control and PUF60-overexpressed OVCAR3 and CAOV3 cells. Scale bar is 10 μm. **d** The number of P-bodies in separate groups of OVCAR8 and ES-2 cells. **e** The number of P-bodies in separate groups of OVCAR3 and CAOV3 cells. **f**-**g** Representative IHC images and the quantitative measurement of DCP1A in subcutaneously transplanted mouse. Scale bar is 50µ. **h**-**i** Representative IHC images and the quantitative measurement of DCP1A and PUF60 in clinical samples. Scale bar is 50µ. Data were presented as means ± SEM. *P < 0.05; **P < 0.01; ***P < 0.001

During our research, we found that colocalization of PUF60 and PABPC1 was observed in cytoplasmic granules, which are highly likely in P-bodies, as shown by DCP1A (a P-body marker) staining (Fig. S3). P-bodies are cytoplasmic ribonucleoprotein (RNP) granules that catalyze mRNA decay occurring throughout the cytoplasm. Hubstenberger et al. purified intact P-bodies and unraveled the P-body proteome [34]. Interestingly, we found that PUF60 and its interaction partner, PABPC1, were the components of P-bodies. We also analyzed the mRNA expression of DCP1A and PUF60/PABPC1 in human OC samples from TCGA and found a positive correlation between the expression of PUF60/PABPC and DCP1A in OC samples (Fig. 7a).

To investigate the role of PUF60 in P-bodies formation, we examined the status of P-bodies in PUF60-knockdown OVCAR8 and ES-2 cells. The results showed that the number of P-bodies was significantly reduced in PUF60-knockdown cells and increased in PUF60-overexpressed cells (Fig. 7b-e), indicating that PUF60 protein is an indispensable protein for P-bodies formation. Furthermore, histological examination of subcutaneous tumors revealed that the DCP1A protein level was decreased in PUF60-knockdown tissues but increased in PUF60-overexpressing tissue (Fig. 7e-f). To further verify the relationship between DCP1A and PUF60, we examined DCP1A and PUF60 proteins in paired clinical samples. The results showed that the expression of DCP1A and PUF60 were both higher in cancer tissue than that in adjacent tissue (Fig. 7f-I, Fig. S5a-d). Above all, PUF60 knockdown decreases the formation of P-bodies in OC.

## Discussion

OC is one of the deadliest malignancies, mainly due to late detection, recurrence, and resistance to conventional chemotherapy. Recent studies show that PUF60 is highly expressed and promotes tumor growth in various types of tumors, in breast cancer, the expression of PUF60 was elevated and its high expression was closely associated with the high incidence of lymph node metastasis and advanced TNM stage, and its upregulation of PUF60 significantly increased the growth, migration, and invasion and repressed the apoptosis through decreased PTEN expression [13]. In bladder cancer cells, the expression of PUF60 was significantly higher in tumor tissues, while high PUF60 expression was associated with malignant phenotypes and shorter survival time, overexpression of PUF60 significantly promoted cell viability and colony formation [9]. In glioblastoma cells, PUF60 is highly expressed and correlated with poor prognosis. PUF60 knockdown significantly decreased the proliferation in vitro and in vivo [35]. In renal cell carcinoma (RCC) cells, PUF60 promote cell growth and the patients with high expression of PUF60 had significantly shorter survival [16]. In our study, PUF60 is highly expressed in OC and its upregulation predicts a poor prognosis, and PUF60 promotes the proliferation and migration of OC cells both in vitro and in vivo, which is consistent with other tumors. However, despite numerous reports that PUF60 promotes tumor growth, its precise mechanism in tumors is remains unknown.

Epithelial OC is a heterogeneous disease consisting of tumors with different types of histologies, grades, and molecular and microenvironmental features, all of which contribute to treatment response and outcome. Histologically, EOC is classified into 5 major subtypes: high-grade serous, low-grade serous, clear cell, endometrioid, and mucinous ovarian cancer [1]. All these subtypes have distinct patterns of presentation and clinical outcomes, as well as responses to therapies. In our study, PUF60 was found mainly in cytoplasm in serous OC and endometrioid OC, but in mainly nucleus in clear cell OC and mucinous OC. The different location may be due to molecular characteristics of subtypes and different transcripts of PUF60. Further studies are needed to prove the speculation.

Our immunohistochemical data showed that the expression of PUF60 is positively correlated with age and patients over 50 years old tend to have higher PUF60 expression. In our country, the mean age at natural menopause of women was 49.3 years [36], during the process of menopause, follicles become atrophic, leading to the decline of estrogen, which consequently promotes the secretion of the gonadotrophin follicle-stimulating hormone (FSH) and luteinizing hormone (LH) from the pituitary gland. According to the ‘gonadotrophin hypothesis’, chronically high levels of FSH may promote the malignant transformation of OSE cells and hence generation of ovarian cancer [36, 37]. Therefore, we speculate that age-related factors, such as hormonal changes or senescence-associated pathways is related to the expression of PUF60, which in turn affects the OC progression.

PUF60 is identified as a splicing factor, and it often conjuncts with U2AF to facilitate the binding of primary transcripts to the U2 snRNP by binding uridine (U)-rich tracts [5]. In some but not all RNAs, PUF60 alone fails to restore the splicing activity in nuclear extracts depleted of poly(U)-binding factors in the absence of U2AF65; thus, U2AF was not strictly required for splicing when PUF60 was present in vitro [5, 8], indicating that PUF60 is not an indispensable protein for alternative splicing. It interacts with RNA polymerase II and the general transcription factor TFIIH, so it is considered to have transcriptional activity [6]. PUF60 also interacts with Ro ribonucleoproteins (RNPs), which are currently thought to play a role in the quality control of small RNAs [38]. Herein, we uncovered a novel function of PUF60 in accelerating mRNA decay and regulating P-body formation in OC, which broadens the previous understanding of its function.

PUF60 belongs to the RNA-binding protein (RBP)family, which is involved in the process of transcriptional and posttranscriptional regulation [39]. The stability of mRNA directly affects the relative expression of cancer driver genes, thereby affecting the occurrence and development of OC. HuR plays an essential role in stabilizing the mRNAs of many tumor-associated genes, such as p53, vascular endothelial growth factor, and c-Fos [40]. CELF2 inhibits OC progression by stabilizing FAM198B [41]. SORBS2 suppresses metastatic colonization of OC by stabilizing tumor-suppressive immunomodulatory transcripts [42]. Here, we discovered that PUF60 promoted the mRNA degradation of oxidative phosphorylation-related target genes, which directly leads to decreased target gene translation, eventually resulting in target gene protein.

Several numbers of studies have shown that RNA-binding proteins are essential for mRNA function. Among them, poly A-binding protein (PABP) is involved in almost all mRNA-dependent events and is accompanied by the entire life process of mRNA synthesis to degradation. There are seven human PABPs, including PABPC1, PABPC3, PABPC4, PABPC4L, ePABP and PABPC5, of which PABPC1 binds poly(A) tails in the nucleus and then transport them to the cytoplasm to complete the first step of mRNA degradation [32]. PABPC1 was upregulated in OC cells and served as a carcinogen to partly promote the OC cell growth and invasion partly by modulating the epithelial–mesenchymal transition [43]. Our data showed that PUF60 interacted with PABPC1, and they had colocalization in OC cells by immunofluorescence. Further study found that PABPC1 interference increased the expression of oxidative phosphorylation related genes, and the cellular functional phenotype is consistent with PUF60 interference. Therefore, we believe that PUF60 interacts with PABPC1 to promote the mRNA degradation of target genes.

Typically, cancer cells adapt to various stress conditions by optimizing gene expression profiles via transcriptional and translational regulation. For example, they regulate translation of some mRNAs by forming mRNPs, such as P-bodies and stress granules in the cytoplasm. P-bodies are cytoplasmic RNA granules that are enriched in the proteins that involved in mRNA decay and translational repression, leading researchers to believe that P-bodies are the site of mRNA decay. Herein, colocalization of PUF60 and PABPC1 was observed in P-bodies, and PUF60 knockdown decreased the formation of P-bodies in OC cells, therefore, we hypothesize that the promotion of mRNA decay by PUF60 may be achieved by promoting the formation of P-bodies.

Abnormal metabolism is a hallmark of cancer [44]. The reprogramming of metabolism is a major trait of the cancer phenotype with great potential for prognosis and targeted therapy [45]. In normal cells, almost all ATP is produced by OXPHOS, while in tumor cells, the production of ATP by OXPHOS is low, but glycolysis is elevated [46]. Cancer cells usually show adaptations to their metabolism that facilitate their growth, invasiveness, and metastasis [47, 48]. OXPHOS downregulation is associated with poor clinical outcomes across several cancer types and it is associated with the presence of epithelial-to-mesenchymal (EMT) signature [49]. Here, we identified that target mRNAs of PUF60 were enriched in OXPHOS, and PUF60 apparently reduced intracellular OXPHOS levels and improved glycolysis levels in OC cells. These metabolic changes could meet the energy demand for the rapid growth of OC cells, indicating that PUF60 might be a key indicator for regulating the energy metabolism of OC cells.

The mitochondrial electron transport chain (Complexes I–IV (CI–IV)) utilizes a series of electron transfer reactions to generate cellular ATP through oxidative phosphorylation. Defects in complex I and III both cause a decline in ATP production by oxidative phosphorylation (OXPHOS) [50]. Mutations in the gene of complex V have recurrently been associated with ATP synthase deficiency [51]. Deficiency in the activity of complex V has been associated with a wide range of human disorders, and is one of the most frequent causes of mitochondrial defects [52]. Assembly of the OXPHOS complexes requires a significant amount of ancillary proteins. In our study, we found 30 of OXPHOS genes were upregulated in PUF60 knockdown cells. Among them, 13 are subunits of Complex I, 8 are subunits of complex V, 5 are subunit of complex III and 4 are subunit of the complex IV. Their high expression of OXPHOS genes was the direct reason for the increase of oxidative phosphorylation level. Therefore, we believe that knockdown of PUF60 improved the level of oxidative phosphorylation by increasing the expression of oxidative phosphorylation genes.

P-bodies are cytoplasmic ribonucleoprotein (RNP) granules comprised primarily of mRNAs in complex with proteins associated with translational repression and 5′-to-3′ mRNA decay, with catalysis of mRNA decay occurring throughout the cytoplasm. Since these factors partition between P-bodies and the cytoplasm, it has remained unresolved whether mRNA decay occurs inside P- bodies or in the cytoplasm [53]. In our study, we found PUF60 and PABPC1 were the components of P-bodies, and we observed that the numbers of P-bodies were reduced in the PUF60-specific knocking down cells, indicating that PUF60 is an indispensable protein for P-bodies formation. So, we speculate that PUF60, along with PABPC1 and other proteins related to mRNA decay, compose P-bodies that speed up mRNA degradation.

Dysregulation of PUF60 could result in abnormal intracellular energy metabolism, therefore, support the malignant state of OC cells. As expected, PUF60 expression was closely correlated with tumor stage and subtype in OC, and highly aggressive subtypes such as clear-cell OC and high-grade serous OC often exhibit higher PUF60 expression levels. The high expression of PUF60 in OC exhibited an oncogenic role, indicating its functional importance in tumorigenesis and the therapeutic potential of OC. Taken together, we report that PUF60 interacts with PABPC1 to promote mRNA decay of OXPHOS genes and the formation of P-bodies, ultimately reducing OXPHOS levels to adapt to the rapid growth of OC cells. This study provides insight into previously unknown functions of PUF60 in RNA processing and suggests that PUF60 may be a novel therapeutic target for OC.

## Conclusions

We uncovered a novel function of PUF60 in accelerating mRNA decay and regulating P-body formation in OC, which broadens the understanding of its function. PUF60 promotes the proliferation and migration of OC cells by regulating cell metabolism, and it may be adopted as a novel therapeutic target.

## Supplementary information

Below is the link to the electronic supplementary material.

ESM 1 (DOCX 15.9 MB)

## Data Availability

All data generated or analyzed during this study are included are available within the paper and supplementary information files.
