# Supplementary material for: Targeting PUF60 prevents tumor progression by retarding mRNA decay of oxidative phosphorylation in ovarian cancer
Source: Cell Oncol (Dordr). 2023 Aug 26;47(1):157–74. doi: 10.1007/s13402-023-00859-w (PMC10899302; doi:10.1007/s13402-023-00859-w)
Supplement: Supplementary file 1 — (DOCX 15.9 MB) [file 13402_2023_859_MOESM1_ESM.docx]

Supplementary Table

Table S1. Primers used in the paper.

|  |  |  |
| --- | --- | --- |
| si-PUF60-1 | sense (5'-3') | GCUACGGCUUCAUUGAGUATT |
|  | antisense (5'-3') | UACUCAAUGAAGCCGUAGCTT |
| si-PUF60-2 | sense (5'-3') | CAGAAAUCAUUGUCAAGAUTT |
|  | antisense (5'-3') | AUCUUGACAAUGAUUUCUGTT |

Table S2. Primers used in the paper.

| Gene | Primer | Gene | Primer |
| --- | --- | --- | --- |
| RPS18-F | ATCACCATTATGCAGAATCCACG | RPS18-R | GACCTGGCTGTATTTTCCATCC |
| PUF60-F | GACCTCTCAGACGATGACATCA | PUF60-R | TCTCGTACTCAATGAAGCCGT |
| ATP5J2-F | ATGGCGTCAGTTGGTGAGTG | ATP5J2-R | TGAAGTCCCGCATCAAGATCC |
| ATP5L-F | ACTACGCCAAGGTTGAGCTG | ATP5L-R | GCCCCGCTTGCCTATAATCTC |
| ATP6V0C-F | ATGTCCGAGTCCAAGAGCG | ATP6V0C-R | GGCCGTAGATGGCGATGAT |
| ATP6V0E1-F | GTCCTAACCGGGGAGTTATCA | ATP6V0E1-R | AAAGAGAGGGTTGAGTTGGGC |
| NDUFS8-F | CCATCAACTACCCGTTCGAGA | NDUFS8-R | CCGCAGTAGATGCACTTGG |
| NDUFA1-F | GCGTACATCCACAGGTTCACT | NDUFA1-R | GCGCCTATCTCTTTCCATCAGA |
| NDUFA2-F | GGGACTTCATTGAGAAACGCT | NDUFA2-R | AGCATTCGCGGATTAGGATGG |
| NDUFA8-F | GCAGGCAAAGTTTGACGAGTG | NDUFA8-R | GATAGGGATTCTCCGGTAAAGGT |
| NDUFC2-F | ACCCAGAACCCTTACGGTTTC | NDUFC2-R | CTCCGCCGGATCAGGTTATC |
| NDUFS5-F | AGTGTTTGCTTCGGCAGAAAA | NDUFS5-R | CCTTCCTTTATCAGCTTATCCCG |
| NDUFS6-F | TTCGGTTTGTAGGTCGTCAGA | NDUFS6-R | CCATCGCACGCTATCACCC |
| COX7C-F | GGTCCGTAGGAGCCACTATGA | COX7C-R | GTGTCTTACTACAAGGAAGGGTG |
| UQCRQ-F | CGCGAGTTTGGGAATCTGAC | UQCRQ-R | TAGTGAAGACGTGCGGATAGG |
| PABPC1-F | CAGGCTCACCTCACTAACCAG | PABPC1-R | GGTAGGGGTTGATTACAGGGT |

Table S3. Antibodies used in the paper.

| Gene | Catalog Number | Applications |
| --- | --- | --- |
| PUF60 | Abcam，ab225705，Rabbit | WB:1/1000, IHC: 1:200 |
| PUF60 | Abcam，ab184538, Mouse | IF: 1:200 |
| PABPC1 | Proteintech, 10970-1-AP, Rabbit | WB:1/1000, IF: 1:100, IP: 4ug |
| DCP1A | Abcam，ab183709，Rabbit | IF: 1:200 |
| Flag | Proteintech, 20543-1-AP, Rabbit | WB: 1/1000, IP: 4ug |
| DCP1A | Santa Cruz，sc-100706，Mouse | IF: 1:20 |
| Ki67 | Servicebio, GB13030, Rabbit | IHC: 1:200 |
| Caspase-3 | Servicebio, GB11009, Rabbit | IHC: 1:200 |
| ATP5J | Proteintech, 14114-1-AP, Rabbit | IHC: 1:100 |
| NDUFA8 | Immunoway, YT3010, Rabbit | IHC: 1:100 |
| NDUFS6 | Abcam, ab195807, Rabbit | IHC: 1:500 |
| NDUFS8 | Abcam, ab170936, Rabbit | IHC: 1:250 |
| β-actin | Hua'an Biology, M1210-2, Rabbit | WB:1/2000 |
| β-Tubulin | Proteintech, 66240-1-Ig, Mouse | WB:1/20000 |

Supplementary Figures

**
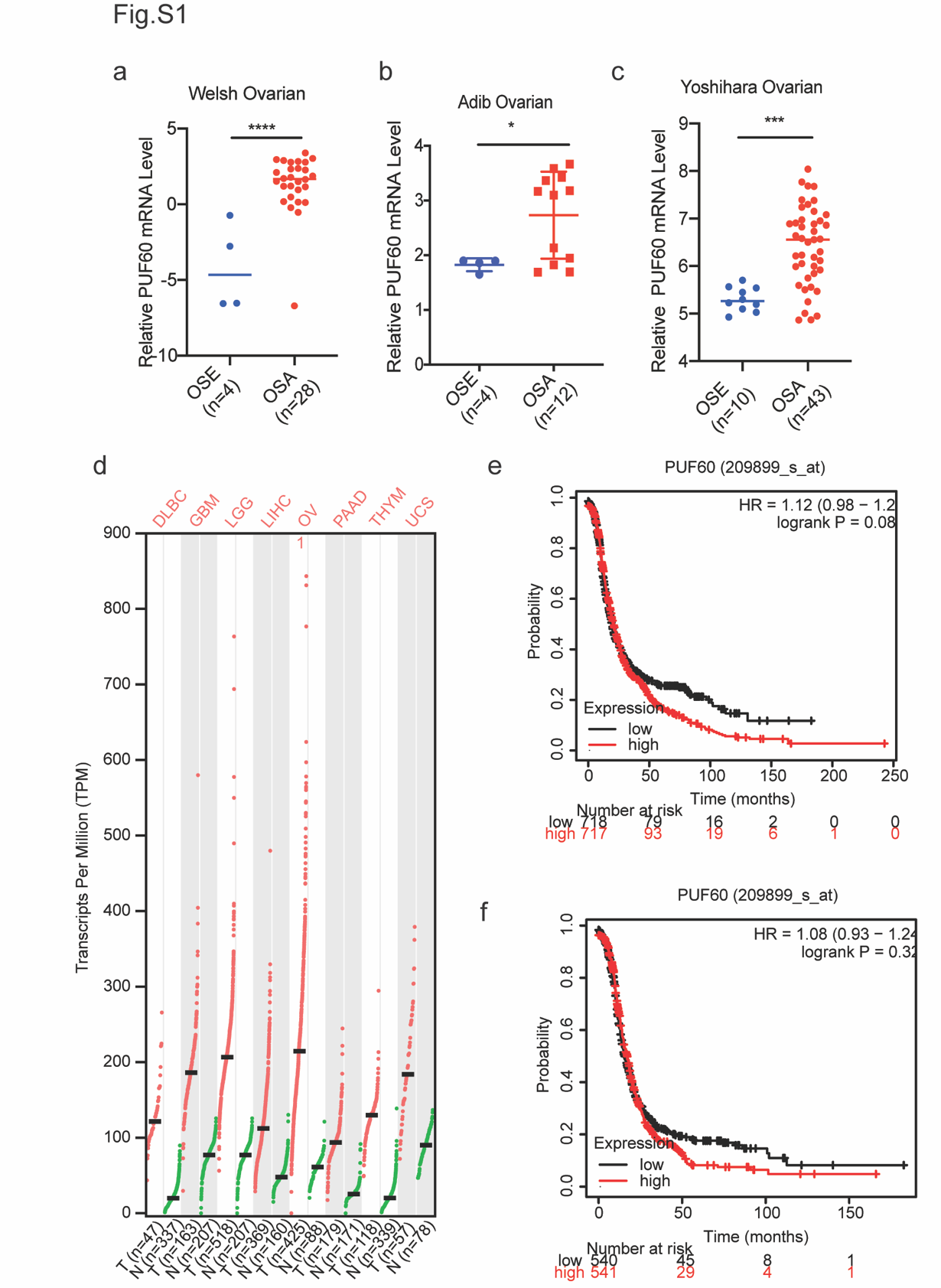
**

**Fig.S1** PUF60 is upregulated in OC. (a-c) PUF60 expression in tumors and normal tissues using Oncomine. OSE: Ovarian Surface Epithelium; OSA: Ovarian Serous Adenocarcinoma. (d) PUF60 expression profile across all tumor samples and paired normal tissues. DLBC: Lymphoid Neoplasm Diffuse Large B-cell Lymphoma, GBM: Glioblastoma multiforme, LGG: Brain Lower Grade Glioma, LIHC: Liver hepatocellular carcinoma, OV: Ovarian serous cystadenocarcinoma, PAAD: Pancreatic adenocarcinoma, THYM: Thymoma, UCS Uterine Carcinosarcoma. (e) Kaplan-Meier survival curve (all stages) of OC data from KM-plotter. (f) Kaplan-Meier survival curve (stage: III+IV) of OC data from KM-plotter. Data are presented as means ± SEM. *P < 0.05; **P < 0.01; ***P < 0.001.


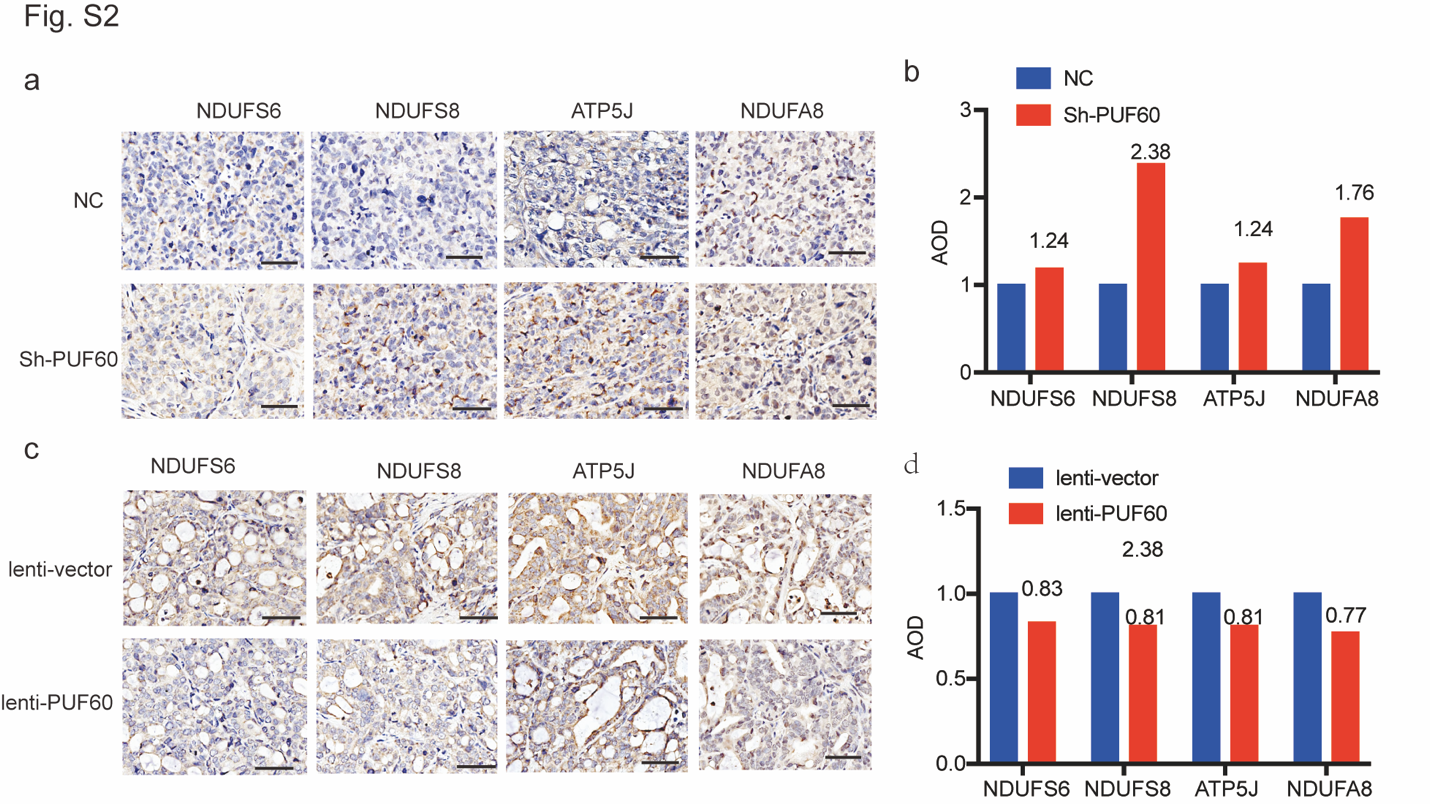


Fig. S2. PUF60 reduces the protein expression of OXPHOS genes. (a-d) Representative IHC images and the quantitative measurement of NDUFS6, NDUFS8, NDUFA8 and ATP5J in subcutaneously transplanted tumor.


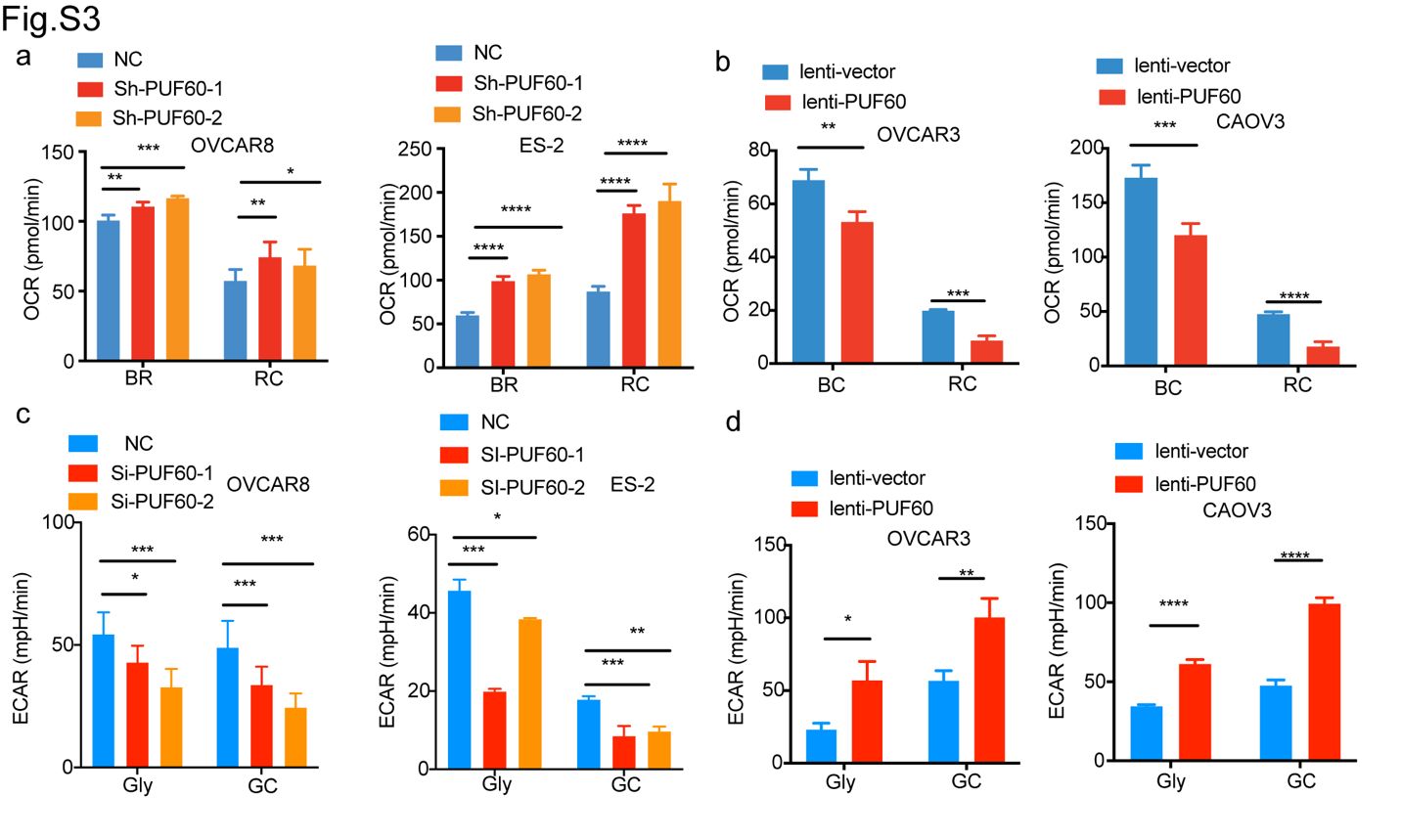


Fig. S3. PUF60 reduces the OXPHOS level and increased glycolysis level in OC cells. (a-b) Basic respiration (BR) and respiratory capacity (RC) of different groups in mitochondrial stress test. (c-d) Glycolysis level (Gly) and glycolysis capacity (GC) of different groups in glycolytic function test.


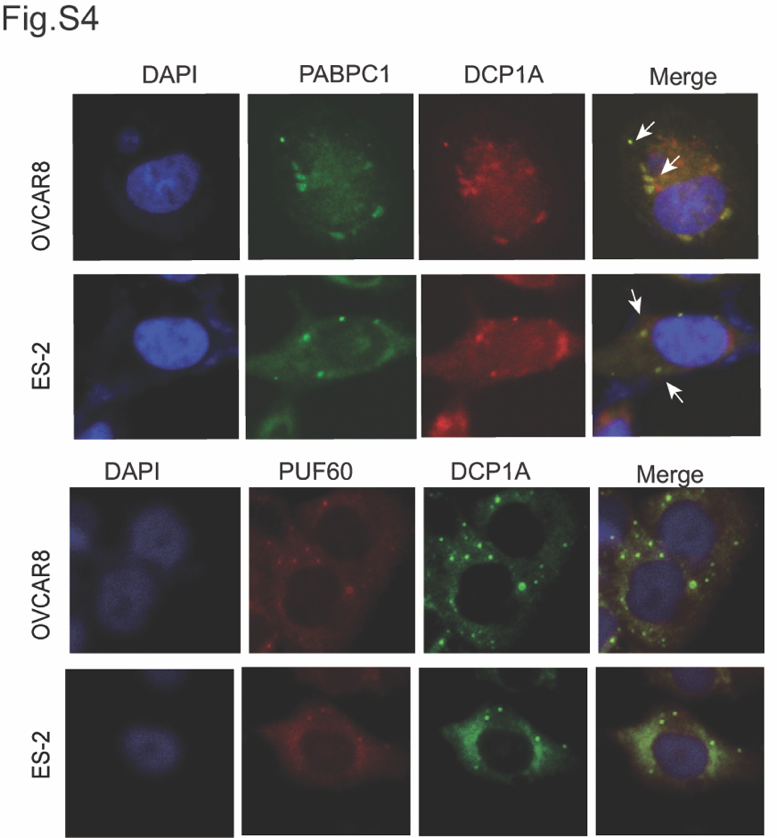


Fig. S4 PUF60 and PABPC1 are localized in P-bodies. Immunofluorescence of PUF60/PABPC1 and DCP1A in control and PUF60-knockdown OVCAR8 and ES-2 cells. Scale bar is 10μm.


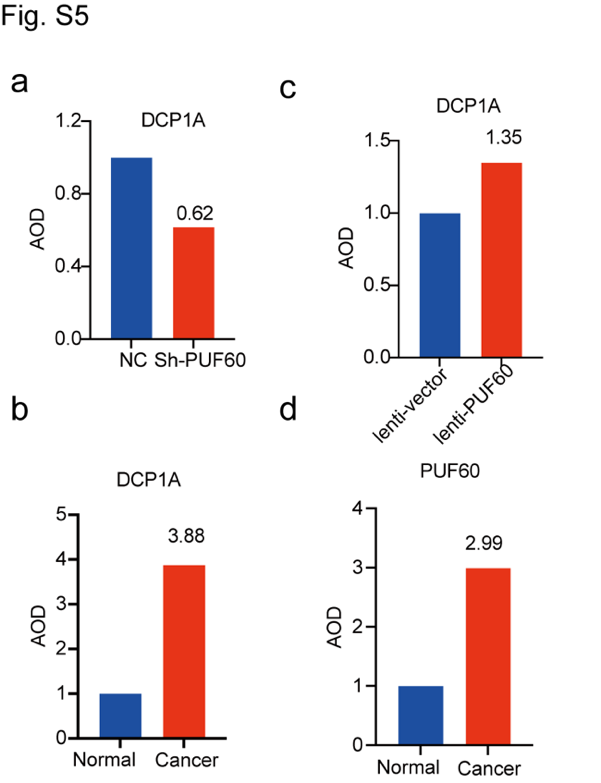


Fig. S5 PUF60 and PABPC1 are localized in P-bodies. (a-d) The quantitative measurement of DCP1A and PUF60 in clinical samples.
